# A Novel Ratiometric Probe Based on Nitrogen-Doped Carbon Dots and Rhodamine B Isothiocyanate for Detection of Fe^3+^ in Aqueous Solution

**DOI:** 10.1155/2016/4939582

**Published:** 2016-03-16

**Authors:** Lin Liu, Lu Chen, Jiangong Liang, Lingzhi Liu, Heyou Han

**Affiliations:** State Key Laboratory of Agricultural Microbiology, College of Science, Huazhong Agricultural University, Wuhan 430070, China

## Abstract

A ratiometric probe for determining ferric ions (Fe^3+^) was developed based on nitrogen-doped carbon dots (CDs) and rhodamine B isothiocyanate (RhB), which was then applied to selective detection of Fe^3+^ in PB buffer solution, lake water, and tap water. In the sensing system, FePO_4_ particles deposit on the surface of CDs, resulting in larger particles and surface passivation. The fluorescence (FL) intensity and the light scattering (LS) intensity of CDs can be gradually enhanced with the addition of Fe^3+^, while the FL intensity of RhB remains constant. The ratiometric light intensity of CDs LS and RhB FL was quantitatively in response to Fe^3+^ concentrations in a dynamic range of 0.01–1.2 *μ*M, with a detection limit as low as 6 nM. Other metal ions, such as Fe^2+^, Al^3+^, K^+^, Ca^2+^, and Co^2+^, had no significant interference on the determination of Fe^3+^. Compared with traditional probes based on single-signal probe for Fe^3+^ detection, this dual-signal-based ratiometric probe exhibits a more reliable and stable response on target concentration and is characterized by easy operation in a simple fluorescence spectrophotometer.

## 1. Introduction

Quantum dots have been extensively investigated for their applications in chemosensor, biosensor, and bioimaging due to their prominent advantages, such as size-dependent fluorescence emission and wide excitation spectrum, whereas narrow fluorescence emission spectrum and high fluorescence quantum yield resistance to photobleaching. Although traditional quantum dots have been widely used in sensor and imaging, their cytotoxicity is still a controversial question [1–4]. In recent years, carbon dots (CDs) [5–8], with low cytotoxicity [9, 10], have attracted extensive research interests due to their good optical properties [5], chemical inertness [11], good biocompatibility [12], and low cost [13]. Due to the above-mentioned advantages, CDs have been widely applied to the development of new methods for detecting ions [14–18], organic molecules [19], and proteins [20].

Traditional fluorescent methods mainly depend on the intensity changes of single-signal fluorescence (increased or decreased). Fluorescence signals of these probes were vulnerable to environment (such as temperature, pH, and viscosity), the influence of the sample itself (such as concentration), equipment effects (such as photobleaching and background light), and other factors. Therefore, strict control of the experimental conditions is beneficial to obtain accurate results. Compared with the single-signal fluorescence probe, ratiometric fluorometry provides an intrinsic correction for external interference. In particular, it can eliminate fluctuations of the excitation light intensity by forming the ratio of the intensity of two well-resolved emission peaks. It is independent of the probe concentration and improves the accuracy of the quantification [21–24].

Herein, we fabricated a dual-signal-based ratiometric probe for the detection of Fe^3+^ and conducted a preliminary test in PB buffer solution. This probe possesses dual emission peaks at 399 nm (CDs) and 577 nm (RhB). The addition of Fe^3+^ to the CD-RhB probe resulted in the rapid increase of FL intensity and LS intensity of the CDs, while the FL intensity of RhB remained constant. By taking advantage of the observed ratios in light intensity between CDs LS and RhB FL, we fabricated a facile ratiometric probe, which can be used to detect different concentrations of Fe^3+^ in both PB buffer solution and actual samples (lake water and tap water).

## 2. Experimental

### 2.1. Chemicals

Ascorbic acid was obtained from Tianjin Kaitong Chemical Reagents Co., Ltd. (Tianjin, China). Polyoxyethylenebis(amine) (PEG-diamine, MW 2000) was purchased from Aladdin Chemistry Co., Ltd. (Shanghai, China). Rhodamine B isothiocyanate was obtained from Sigma-Aldrich. NaCl, KNO_3_, Fe_2_(SO_4_)_3_·7H_2_O, Zn(NO_3_)_2_·6H_2_O, CoCl_2_·6H_2_O, AgNO_3_, Al_2_(SO_4_)_3_·18H_2_O, CaCl_2_·2H_2_O, CdCl_2_·2.5H_2_O, CuSO_4_·5H_2_O, and FeSO_4_·7H_2_O were acquired from Shanghai Chemical Reagent Co., Ltd. Dialysis bag (MWCO: 1000) was purchased from Aladdin Chemistry Co., Ltd. All reagents were used without further purification and all the solutions were prepared using ultrapure water obtained from a Millipore water purification system (Milli-Q, Millipore, 18.2 MΩ resistivity).

### 2.2. Apparatus

All fluorescence measurements were carried out with RF-5301PC fluorescence spectrometer (Japan); the sample was placed in a 10 mm quartz fluorescence cuvette. The UV-Vis absorption spectra were recorded between 200 and 700 nm on a UV-2450 (JAPAN) with a 1.0 cm path-length cuvette. High-resolution transmission electron microscopy (HRTEM) (JEM-2100F, JEOL) was used to characterize the size and surface morphology of the as-prepared CDs. Fourier transform infrared spectroscopy (FT-IR) was conducted to detect the chemical identity of CDs on a Nicolet Avatar-330 spectrometer (Thermo Nicolet, USA) with 4 cm^−1^ resolution using the KBr pellet technique. All Raman spectra were recorded at room temperature using a Raman spectrometer (Renishaw, UK) equipped with a confocal microscope (Leica, German). Flame atomic absorption spectrometry (FAAS) was obtained with AA240FS spectrometer (Varian, Inc., USA) to detect the content of Fe^3+^ in the actual samples. All pH measurements were carried out with a PHS-3C pH Meter (Shanghai, China).

### 2.3. Synthesis of the CDs

The CDs were synthesized by a hydrothermal method according to the literature with some modifications [25]. 0.10 g polyethylene glycol diamines and 0.20 g ascorbic acid were mixed and ground sufficiently in an agate mortar. Then, the mixture was transferred to a 25 mL hydrothermal reaction kettle and heated for 1 h at 180°C to get a dark brown mixture that was dissolved with 5.0 mL ultrapure water. Subsequently, the mixture was centrifuged at 12000 rpm for 10 min. The supernatant was collected and then dialyzed against ultrapure water through a dialysis membrane for 24 h to obtain the pure CDs.

### 2.4. Preparation of Carbon Nanoprobes

The preparation of carbon nanoprobes followed Huimin Ma's method [26]. The as-prepared CDs solution above was mixed with RhB at a 4 : 1 mass ratio in 0.10 M NaHCO_3_ solution at room temperature overnight. Then, the reaction solution was dialyzed in 0.10 M NaHCO_3_ and ultrapure water every 24 h by a membrane with a molecular weight cutoff of 1000. After gel chromatographic separation on a Sephadex G-100 column with water as eluent, the product was collected and stored at 4°C for further use.

### 2.5. Characterization by Raman Spectroscopy

The specific experimental procedures were as follows. 10 *μ*L of the probe was added into 950 *μ*L PB buffer solution (10 mM, pH 7.4) and was mixed to uniformity. Then, 40 *μ*L of 30 *μ*M Fe^3+^ was added dropwise into the above solution, and the mixture was centrifuged for 30 min at 12000 rpm. To completely remove the interference of phosphate radical, the supernatant was removed and the precipitate was washed with ultrapure water for three times. Then, ultrapure water was added and the precipitation was dissolved by ultrasound. The obtained liquid dropped on the foil. Raman spectroscopy was recorded at the excitation wavelength of 633 nm until the liquid was dried.

## 3. Results and Discussion

**Figure 1:**
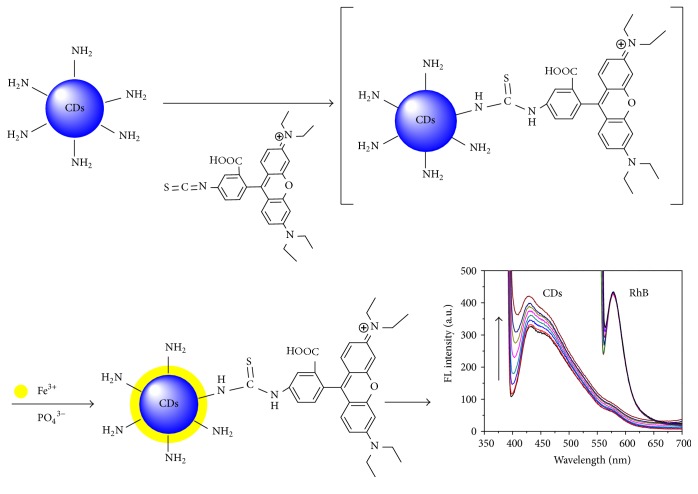
TEM image (a) and size distribution (b) of CDs. (c) UV-Vis absorption spectrum and FL intensity spectrum of CDs (inset shows the photographs of CDs under day light and ultraviolet light). (d) FL emission spectra (with progressively longer excitation wavelengths from 340 to 400 nm) of CDs.

High-resolution transmission electron microscopy (HRTEM) was used to characterize the surface morphology of the as-prepared CDs. The CDs are well dispersed in aqueous solution and their diameters range from 2.7 to 5.9 nm, with a mean diameter of 4.4 ± 0.6 nm (Figures 1(a) and 1(b)). The UV-Vis absorption spectrum and the fluorescent spectrum were given in Figure 1(c). Due to the *n*-*π* ^*∗*^ transition of CDs, the resultant CDs displayed a broad UV-Vis absorption [9, 25]. In addition, the UV-Vis absorption spectrum shows a strong peak at 249 nm, which could be ascribed to the *π*-*π* ^*∗*^ transition of aromatic sp^2^ domains [27, 28]. It can be clearly observed that the most intense peak of CDs appears at 450 nm (emission wavelength) with excitation at 370 nm. Therefore, CDs show blue color under UV (365 nm) light (Figure 1(c) inset), with the obtained CDs with a quantum yield of 15%. Moreover, the corresponding FL emission spectra of CDs were plotted with the excitation wavelength progressively increasing from 340 to 400 nm (Figure 1(d)). It is obvious that the FL emission peak of CDs exhibits a large red shift (from 420 nm to 480 nm) with an increase of the excitation wavelength, and the FL intensity increases until *λ*ex = 370 nm, and then decreases slowly. The result that the emission of CDs strongly depends on the excitation wavelength is consistent with the results reported in previous studies [25, 29].

**Figure 2:**
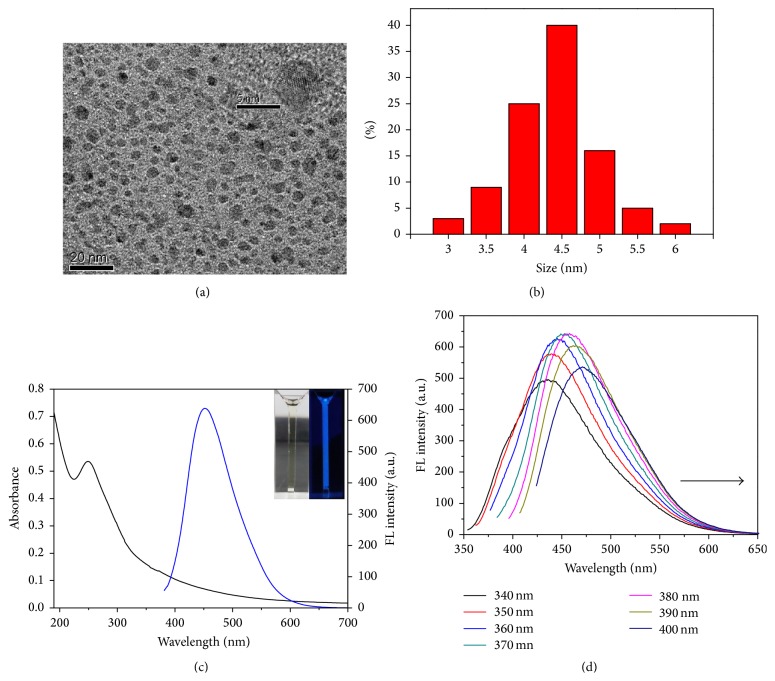
FL intensity of CDs in aqueous solutions with different pH values. All values were obtained based on three independent measurements.

**Figure 3:**
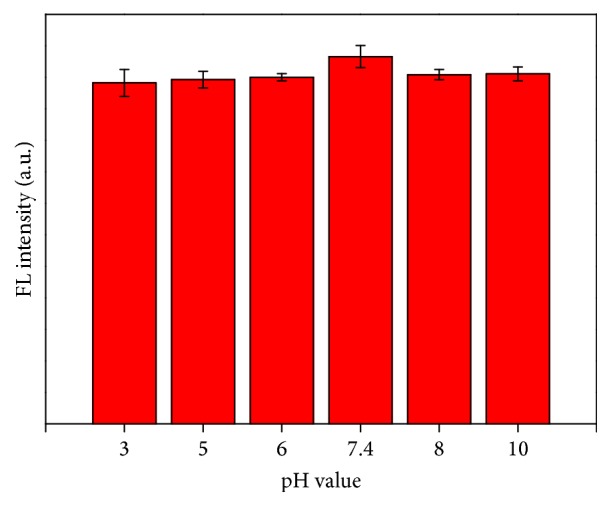
FT-IR spectra of the CDs.

The effects of pH on emission stability of CDs were monitored in 10 mM PB buffer solution. It is clearly shown in Figure 2 that the FL intensity of CDs nearly has no change at pH ranging from 3.0 to 10.0, indicating that CDs can work in environments at a wide range of pH values. Fourier transform infrared (FT-IR) spectra were used to identify the functional groups present on the surface of the as-prepared CDs: stretching vibrations of C-OH at 3409 cm^−1^ and C-H at 2901 cm^−1^; the peak at 1350 cm^−1^ from the stretching vibration of C-NH, which indicates the successful adulteration of nitrogen atoms into the CDs; bending vibrations of N-H at 1685 cm^−1^; the vibration absorption band of C=O at 1762 cm^−1^ and the peaks at 1042 and 1105 cm^−1^ related to the C-OH stretching vibrations, which imply the oxygen-rich property of the CDs (Figure 3) [5, 25]. These functional groups improve the hydrophilicity and stability of the as-prepared CDs, suggesting their great advantage to be used as sensor in aqueous solution.

**Figure 4:**
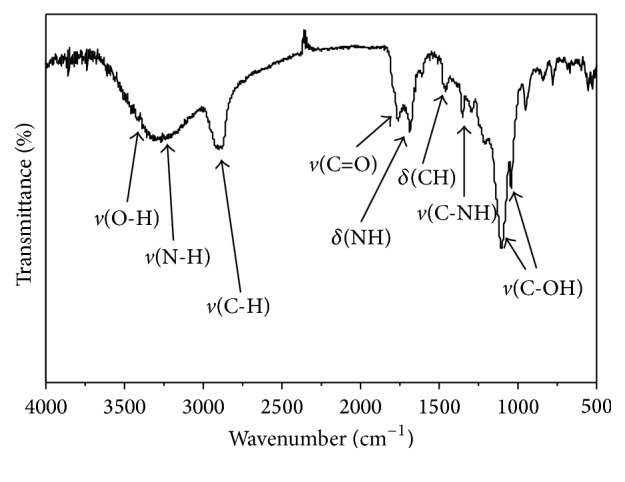
(a) FL emission spectra of the CD-RhB probe with different concentrations of Fe^3+^ (from bottom to top, 0, 0.01, 0.15, 0.3, 0.6, 0.9, and 1.2 *μ*M) in PB buffer solution (10 mM, pH 7.4). (b) The *I*_399_/*I*_577_ ratios of the probe versus the concentration of Fe^3+^ within the range of 0.01–1.2 *μ*M. The error bars represent standard deviations based on three independent measurements.

According to previous reports, doping CDs with trace impurities enables the alteration or an increase in the number of emission centers [30, 31]. Nitrogen-doped CDs are the most widely studied doped CDs that exhibit both defect-related emission and scattering emission. Figure S1 in Supplementary Material available online at http://dx.doi.org/10.1155/2016/4939582 indicates that the LS peak of CDs at 399 nm was observed after quench of FL. The defect-related emission leads to FL in a wavelength range of 400–700 nm. Whilst, nitrogen-doped CDs are capable of scattering the incident light and the LS intensity can be greatly increased upon particle aggregation [32]. This probe possesses distinguished advantages over other probes for the LS resulting from target-induced aggregation of nanoparticles [33, 34]. RhB was selected as the reference signal in the probe due to its chemical inertness in the presence of Fe^3+^. As shown in Figure S2, the hydrodynamic size distribution of CD-RhB probe was increased after the addition of Fe^3+^.  Figure 4(a) presents the emission spectra of RhB upon the addition of Fe^3+^. It is clear that the FL spectra remain almost unchanged with the addition of Fe^3+^; in contrast, when the concentration of Fe^3+^ ranges from 0.01 to 1.2 *μ*M, the light intensity of CDs increases in the presence of Fe^3+^, especially the intensity of LS. Based on these findings, we expect that the probe can selectively detect Fe^3+^ with high sensitivity. As shown in Figure 4(a), the LS intensity of CDs shows a rising trend and the FL intensity of RhB basically remains unchanged in the presence of different concentrations of Fe^3+^. The ratios in light intensity between CD LS and RhB FL (*I*_399_/*I*_577_) display a good linear relationship with the Fe^3+^ concentrations ranging from 0.01 to 1.2 *μ*M with a detection limit of 6 nM under the optimum experimental conditions (Figure 4(b)). The linear equation is *I*_399_/*I*_577_ = 0.256 + 0.379*c* (*c*: *μ*M) and the correlation coefficient *R* ^2^ is 0.996, where *I*_399_ and *I*_577_ are the light intensities at 399 nm and 577 nm in the absence or the presence of Fe^3+^, respectively.

**Figure 5:**
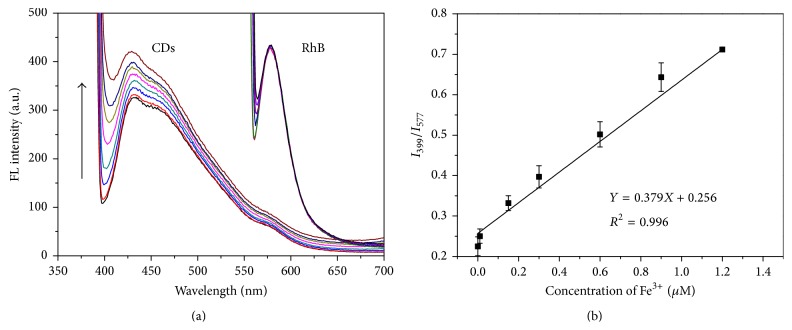
Selectivity of the CD-RhB probe toward Fe^3+^ over other ions. The concentration of metal ions is 1.0 *μ*M. The error bars represent standard deviations based on three independent measurements.

The specificity of the CD-RhB probe for Fe^3+^ with a variety of metal ions including Fe^2+^, K^+^, Co^2+^, Zn^2+^, Al^3+^, Cu^2+^, Ag^+^, Na^+^, Ca^2+^, and Cd^2+^ was evaluated in PB buffer solution (10 mM, pH 7.4). The *I*_399_/*I*_577_ ratios of different metal ions at the same concentration (1.0 *μ*M) were evaluated in PB buffer solution. As shown in Figure 5, there is no significant interference on the probe from the above cations. This result indicates that our CD-RhB probe exhibits a high specificity for Fe^3+^ over other metal ions.

## 4. Possible Mechanism by Which Fe^**3+**^ Affects Probe Light Intensity

**Scheme 1:**
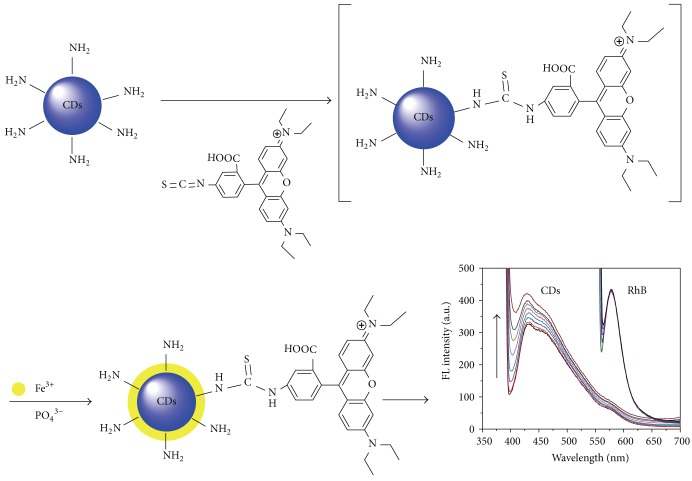
Dual-signal ratiometric probe of Fe^3+^ based on a CD-RhB nanohybrid system.

**Figure 6:**
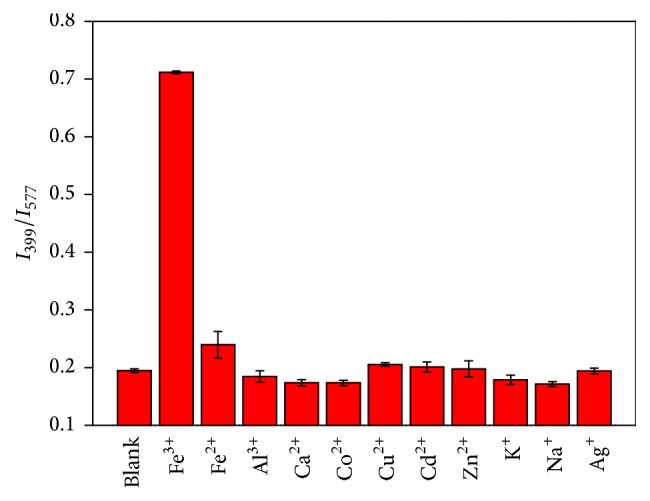
Raman spectra of FePO_4_ collected as a function of power. The wavelength of the excitation laser is 633 nm.

We constructed CD-RhB probe through the conjugation of CDs and RhB molecules. With the addition of Fe^3+^, the FL and LS of CDs in the probe were gradually enhanced, while the FL of the RhB remained constant. Therefore, we propose a working mechanism of the FePO_4_-mediated LS and FL increase of CDs: while FePO_4_ particles are deposited on the surface, the defects of the CDs are filled, and thus the LS intensity can be greatly increased along with the appearance of larger particles. In addition, the interactions of FePO_4_ particles with CDs result in surface passivation of the CDs, thereby enhancing the FL intensity (Scheme 1). Then, the hypothesis was verified by Raman spectroscopy. As shown in Figure 6, a very prominent peak appears at 1003 cm^−1^, which indicates the presence of FePO_4_ according to the previous study [35]. Except for the peak of FePO_4_, no responses were observed for CDs, RhB, or CD-RhB nanohybrid on the whole Raman spectrum.

## 5. Calibration and Application in Actual Samples

To demonstrate the practicability of the ratiometric probe, its detection performance was evaluated in actual water samples. The proposed method was successfully applied to determine Fe^3+^ content in lake water (sample 1) and tap water (sample 2). The lake water was taken from South Lake (Wuhan), and the tap water was collected from our laboratory. After the environmental water samples were centrifuged at 6000 rpm for 30 min and then filtered through 0.22 *μ*m filter membranes, they were used for Fe^3+^ analysis. Parallel measurements were carried out with three similar water samples by FAAS. The results obtained by the proposed method are in good consistency with those of FAAS (given in Table 1). To further validate the determination, addition and recovery of Fe^3+^ in actual water samples were also studied, and the obtained recoveries ranged from 97.2% to 108.8% (Table S1 in the Supporting Information).

## 6. Conclusion

In summary, the as-prepared nitrogen-doped CDs are stable, bright, and with good water solubility. We have demonstrated that CDs and RhB form a nanohybrid probe through their chemical reaction. This CD-RhB probe was successfully used as a ratiometric probe to detect Fe^3+^ in lake water and tap water, showing the good selectivity and sensitivity. The detection limit is as low as 6 nM. Compared with the previously reported sensing methods, this CD-RhB probe displays several important advantages. First, this strategy avoids the use of traditional semiconductor quantum dots and organic solvents; thus, it is more environmental friendly. Second, the ratiometric probe can effectively eliminate the background interference and the fluctuation of detection conditions by depending on two kinds of signals: CDs LS and RhB FL, and thus it is more reliable than single-signal detection strategy. Third, this ratiometric probe for Fe^3+^ determination exhibits more sensitive signals than quenching probe. It is expected that this strategy may offer a new approach for developing green, low-cost, and sensitive dual-signal probes for practical applications.

## Supplementary Material

The supplementary data contains the experiment description in the assay of Fe3+, the measure-ment of quantum yield of CDs, the fluorescence curves of CDs under xenon lamp, the hydrodynamic size distribution of CD-RhB probe before and after adding Fe3+ and the standard addition experiments of Fe3+ in actual water samples.

## Figures and Tables

**Table 1:** Determination of Fe^3+^ in lake water and tap water with the proposed method.

Sample	Proposed method (*μ*M)	FAAS (*μ*M)
Sample 1 (lake water)	0.036 ± 0.006	0.044
Sample 2 (tap water)	0.060 ± 0.009	0.065
