# Supplementary material for: A Novel Ratiometric Probe Based on Nitrogen-Doped Carbon Dots and Rhodamine B Isothiocyanate for Detection of Fe3+ in Aqueous Solution
Source: J Anal Methods Chem. 2016 Mar 16;2016:4939582. doi: 10.1155/2016/4939582 (PMC4826956; doi:10.1155/2016/4939582)
Supplement: Supplementary file 1 — The supplementary data contains the experiment description in the assay of Fe3+, the measure-ment of quantum yield of CDs, the fluorescence curves of CDs under xenon lamp, the hydrodynamic size distribution of CD-RhB probe before and after adding Fe3+ and the standard addition experiments of Fe3+ in actual water samples. [file 4939582.f1.pdf]

Supporting Information

Experimental Section

General Procedure of Fluorescence Assay of Fe^{3+}. In a typical run, the solutions of the PB buffer (1 mL, 10 mM, pH 7.4) and CD-RhB probe dispersion solution (20 μL) were sequentially added into a 2 mL centrifugal tube. Then, Fe^{3+} solution was added to give a series of samples with different concentrations (range, c = 0 - 1.2 μM). The resultant solution was mixed thoroughly. After incubation for 2 min, the FL spectral data of the resultant solution were collected over the wavelength range of 350 - 700 nm. All measurements were taken at room temperature.

Measurement of quantum yield of CDs. The quantum yield of the CDs was calculated using following function [S1]:

$$\Phi = \Phi_R \times \frac{I}{I_R} \times \frac{A_R}{A} \times \frac{\eta^2}{\eta_R^2}$$

Quinine sulfate in 0.1 M H_2SO_4 (literature quantum yield 0.54 at 360 nm) was chose as a standard. Where Φ is the quantum yield of the sample, I is the measured integrated emission intensity, η and A is the refractive index and optical density, respectively. The subscript R refers to the reference fluorophore of known quantum yield. In order to minimize reabsorption effects, absorption in the fluorescence cuvette was kept below 0.10. The obtained CDs with a quantum yield of 15%.

The LS peak of CDs. We irradiated continuously the diluted CDs solution under xenon lamp for six hours. The LS peak of CDs at 399 nm was observed when the FL peak of CDs was quenched.

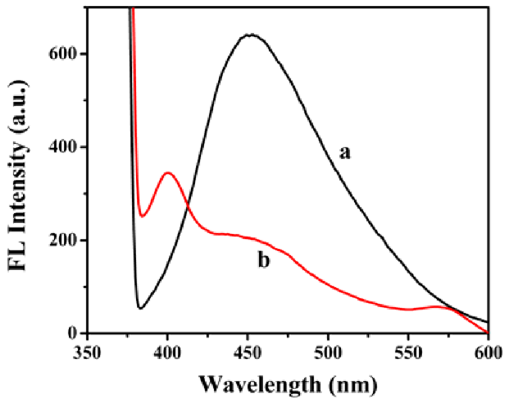

Figure S1. The spectra of the CDs before (a) and after (b) irradiation under xenon lamp for six hours.

Dynamic light scattering characterizations of CD-RhB probe before and after adding Fe^{3+}

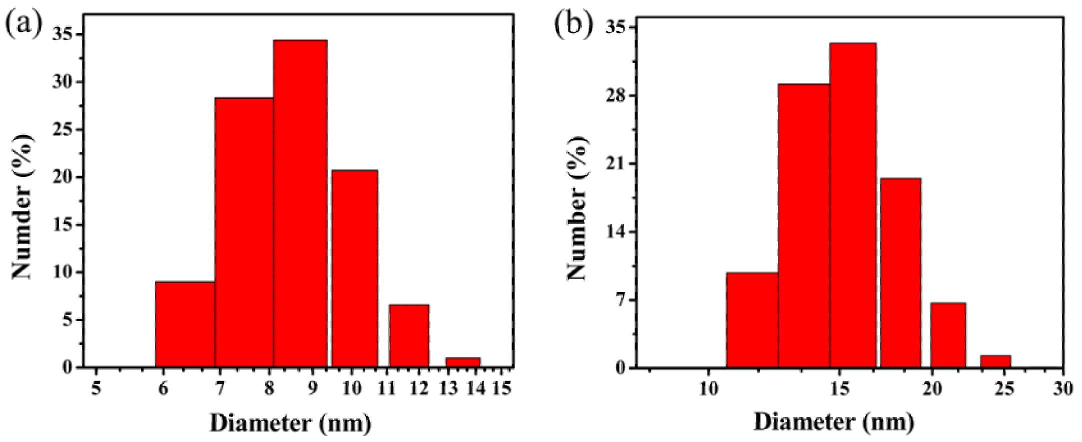

Figure S2. The hydrodynamic size distribution of CD-RhB probe before (a) and after (b) adding Fe^{3+} by dynamic light scattering.

Determination of Fe^{3+} in actual water samples. To further validate the determination, standard addition experiments were performed with the actual water samples. For the recovery study, two fortification levels were assayed, and the recoveries values were shown in Table S1. The obtained recoveries ranged from 97.2% to 108.8% depending on the concentration level.

Table S1. Analytical results of the determination of Fe^{3+} in lake water and tap water samples using the proposed dual-signal ratiometric probe.

Sample	Found Fe^{3+} in sample (μM)	Fe^{3+}		Recovery (%)
		Added (μM)	Total found (μM)	
Sample 1 (lake water)	0.036 ± 0.006	0.50	0.527	98.2
		1.00	1.039	100.3
Sample 2 (tap water)	0.060 ± 0.009	0.50	0.604	108.8
		1.00	1.034	97.4

References

S1. Y. Guo, B. X. Li, “Carbon dots-initiated luminol chemiluminescence in the absence of added oxidant,” Carbon, 82: 459-469, 2015.
